# Effects of season and age at first calving on genetic and phenotypic characteristics of lactation curve parameters in Holstein cows

**DOI:** 10.1186/s40781-016-0089-1

**Published:** 2016-02-22

**Authors:** Mahdi Elahi Torshizi

**Affiliations:** Department of Animal Science, Mashhad Branch, Islamic Azad University, Mashhad, Iran

**Keywords:** Age at first calving, Lactation curve parameters, Correlation, Heritability, Breeding value

## Abstract

In this research data representing 72,946 primiparous cows from 724 herds with 638,063 total test day records calved between 2001 and 2011. These data were analysed to determine the effect of age at first and season of calving on parameters of the Wood lactation curve. Also, genetic trend of the lactation curve parameters in different calving years were evaluated. The results indicate that the highest rate of atypical lactation curve was related to cows that calved in summer (28.05 %). The maximum phenotypic relationship between initial milk yield and total 305-d milk yield was observed in cows calved in spring (0.40). The role of peak yield is more than peak time on 305-d total milk yield in primiparous Holstein. One month increase in age at first calving from 18 to 26 month raised 305-d milk yield by around 138 kg and from 27 to 32 month decreased by 61 kg. The persistency of lactation between 101 and 200 days is higher than that of 201–305 days. Our results indicate that the shape of lactation curve is largely dependent on the season of calving (higher level of milk production in cows which calved in autumn and winter). The heritabilities of parameters of lactation curve and persistency measures were low. The genetic trends for peak time, peak yield and 305-d milk yields were positive and estimated to be 0.019, 0.021 and 8.13 kg/year respectively. So the range from 24 to 26.5 month of calving is the optimum calving time in primiparous Holstein for maximizing 305-d milk yield.

## Background

Age at first calving is extremely important economic trait determining the profit of cow milk production. Milk yield has a great economic impact on dairy cattle industry and on the level of income in small and large farms especially in developing countries. Daily milk yield and milk components are being changed continuously during lactation. Lactation curve is the graphical representation of the relationship between milk yield and lactation length. Daily milk yield and milk components are influenced by genetic and environmental effects [1]. The genetic effect is referred to differences in milk production and composition among breeds or genotypes and individuals within each breed. The most important environmental or non-genetic effects are the stage of lactation [1], age at calving, parity, calving season [2, 3], pregnancy stage [4] and common herd effect. Reproductive performance is the trait of outstanding importance in dairy cattle enterprises, where if there is no calf, then there is no economic return. So, one of the most important factors influence on reproductive performance of the females are age at first calving (AFC) and season of calving [5]. Several results have reported reduction of age at first calving in dairy cattle. Age at calving is more important in earlier lactations [6]. Pirlo et al. [5] showed that decreasing age at first calving has positive effect on genetic progress. Also, Niloforooshan and Edriss [7] who studied on Iranian Holsteins, found that the optimum age at first calving to maximize first lactation ME milk yield, was 24 months [8]. They suggested that the optimum AFC in Holsteins for maximum profit should be 23 to 24 months of age. Moreover, reducing age at first calving can reduce feed costs and increase the number of calves per cow. But Pirlo et al. [5] mentioned that early calving is harmful to milk yield and longevity. Moreover, it has negative effects on first lactation milk yield and fat percentage as well. For USA dairy cattle, Hare et al. [9] reported decreased trend for age at first calving from 1980 to 2004. Dobos et al. [10] showed that heifers calving at younger AFC produce similar amounts to their older herd-mates by the end of the third lactation. In the statistical models, age at calving can be modelled as linear [11], quadratic regression [12] or treated as fixed class effect [13, 14]. Calving season referrers to that season of the year when cow calves and starts with lactation period. Seasons with high temperatures have negative influence on milk traits, mainly fat content. Calving season causes differences in milk production and in the shape of lactation curve. Several studies [2, 15] showed that cows which calved during autumn or winter had higher milk yield in comparison to cows calved in spring or summer. The lactation curve of cows which calved in spring had a typical shape, with a peak followed by a continuous decline [16]. Autumn calving cows had higher yield at mid and late lactation. Moreover, lactation starting in summer and autumn are more persistent than lactations starting in winter or spring [17]. They also reported that for every month of increase in age at first calving, total milk yield increased by 28 kg of milk. Bouallegue et al. [18] reported that the lowest level of production occurred for cows calved in summer, but these cows are more persistent. The objectives of this study are evaluation of age at first calving and calving seasons on ratio of atypical lactation curve and also lactation curve parameters in Iranian Holstein cows. Also genetic trends of lactation curve parameters in different calving year using animal model analysis were calculated.

## Methods

A total of 638,063 test day milk yield of dairy cows calved from 2002 to 2011 were analysed. Cows age at calving varied from 18 to 32 months, The numbers of test day records per cows were from 3 to 10 and cows with daily milk production lower than 2 kg were discarded from data. The structure of data set and pedigree after editing are summarized in Table 1.

**Table 1:** Description of the database

No. of cows	72,946	No. of records	638,063
No. of sire	2584	No. of herd	724
No. of founders	51,612	No. of non-founders	72,949
No. of dam	61,867	Average No. progeny/sire	28.22
Year of calving	2002–2011	Average No. progeny/dam	1.17
Age at calving:		Milk yield:	
Average (month)	24.35	Average (kg)	29.12
^a^SD (month)	1.38	SD (kg)	7.07
Maximum (month)	32	Maximum (kg)	75.6
Minimum (month)	18	Minimum (kg)	2

^a^Standard deviation

The individual lactation curve was fitted using the Wood function and parameters of the lactation curve were estimated by Nlin procedure of the statistical program package SAS [19]. The Wood model and its by-product parameters have proved powerful on fitting test day data in the tropical and sub-tropical regions [20, 21]. These parameters determined the shapes of lactation curves. The Wood mathematical model was as follow:

$$ {\mathrm{Y}}_{\mathrm{t}}=\mathrm{a}{\mathrm{t}}^{\mathrm{b}}{\mathrm{e}}^{\hbox{-} \mathrm{c}\mathrm{t}} $$

Where

t = days in milk

Y_t_ = milk yield in kg

a = approximates the initial milk yields after calving

b = the inclining slope parameter up to yield peak

c = the declining slope parameter

The days at peak milk yield, Peak milk yield, and total milk of 305-d were calculated as b/c, a(b/c)^b^exp^‐ b^ and ${\displaystyle \sum_{\mathrm{i}=5}^{305}\mathrm{a}{\mathrm{t}}^{\mathrm{b}}\mathrm{ex}{\mathrm{p}}^{\hbox{-} \mathrm{c}\mathrm{t}}}$ respectively. Moreover, partial productions from days 5–100 (part1), 101–200 (part2), 201–305 (part3) were calculated individually for each animal. Atypical lactation curves, with negative values for b or c were excluded and distribution of typical and atypical lactation curves according to season and month of calving were calculated. Total of 72,946 of the first lactation records were used (18,160 in spring, 19,058 in summer, 17,634 in autumn and 18,094 in winter) and number of cows in different months of calving were 124 in 19 month, 270 in 20 month,742 in 21 month, 2537 in 22 month, 9561 in 23 month, 2854 in 24 month, 19,547 in 25 month, 13,124 in 26 month, 4526 in 27 month, 1360 in 28 month, 500 in 29 month, 225 in 30 month and 42 in 31 month respectively. Based on partial yields, different measures of persistency were used in literature. In this study two different measures of persistency (persis1, persis2) were used. Persis1 was calculated as the ratio of milk yield between 101 and 200 days after parturition to milk yield in the first 100 days of the lactation and persis2 was calculated as the ratio of milk yield between 201 and 305 days after parturition to milk yield in the first 100 days of the lactation. The effect of environmental factors on dependent variables (a, b, c, peak time, peak yield, part1, 2, 3, milk 305-d, persis1 and persis2) were investigated by analysis of variance (PROC GLM) with the following model:

$$ {\mathrm{Y}}_{\mathrm{nijkcpl}}=\upmu +{\mathrm{H}}_{\mathrm{i}}+\mathrm{C}{\mathrm{Y}}_{\mathrm{j}}+\mathrm{P}{\mathrm{Y}}_{\mathrm{k}}+{\mathrm{S}}_{\mathrm{c}}+{\mathrm{S}}_{\mathrm{p}}+\mathrm{A}\mathrm{F}{\mathrm{C}}_{\mathrm{l}}+{\mathrm{e}}_{\mathrm{nijkcpl}} $$

where Y_nijkcpl_ is dependent variables on n^th^ animal affected by i^th^ herd (724 herd), j^th^ calving year (10 years from 2002 to 2011), k^th^ year of milk production, c^th^season of calving, p^th^ season of milk production, l^th^ age at first calving class (15 months from 18 to 32) and e_mijkcpl_ is the random effect of residuals with expectation and variance equal to 0 and σ_e_^2^ respectively. μ is the overall mean, H_i_, CY_j_, PY_k_, S_c_, S_p_ and AFC_l_ are effects of herd (located in semi-arid area), calving year, milk production year, season of calving (April through June (spring), July through September (summer), October through December (autumn) and January through march (winter), season of milk production and age at first calving, respectively. Phenotypic trends of parameters of lactation curve were calculated using least square means of phenotypic values on age at calving. Genetic data analyses were done by simple univariate animal model using a restricted maximum likelihood method of WOMBAT program [22]. The model was:

$$ \mathrm{y}=\mathrm{X}\upbeta +\mathrm{Z}\mathrm{a}+\mathrm{e} $$

Where y were a vector of dependent variables, β is a vector od fixed effects, a is a vector of additive genetic values of animals, e is a vector of residual effects, X and Z are incidence matrices for fixed and random effects. E(y) = Xb; E(a) = E(e) = 0. Genetic trends of the lactation parameters were obtained by regressing means estimated breeding value on calving year.

## Results and discussion

The distribution of typical and atypical lactation curve in our dataset, based on season and age at calving, are presented in Tables 2 and 3, respectively. In this study a lactation curve was considered atypical if b or c was negative. The effects of calving season and age at calving were significant of atypical lactation curve. The frequency of atypical lactation curve ranged from 19.92 % in autumn to 28.05 % in summer (Table 2). Based on this result the highest and lowest frequency of atypical lactation curve were observed in spring & summer and autumn & winter, respectively. An effect of calving season on occurrence of atypical lactation yield has been reported by Rekik et al., [20] and Cilek et al. [23]. Also, Macciotta et al. [24] reported that the occurrence of atypical lactation curve is the highest in cows which calved during summer. As proposed by these authors, it seems that the health troubles related to severe environmental conditions like heat stress as well as biological conditions of the animals in summer lead to atypical lactation curve.

**Table 2:** Distribution of typical and atypical lactation curves according to season of calving

Atypical lactation curve					
Calving season	Negative B	Negative B and C	Total	No. typical curve	X^2^
Spring	2994	1893	4887(26.91 %)	13,273(73.09 %)	–
Summer	3191	2156	5347(28.05 %)	13,711(71.95 %)	–
Autumn	2160	1354	3514(19.92 %)	14,120(80.08 %)	–
Winter	2366	1430	3796(20.97 %)	14,298(79.03 %)	–
Total	10,711	6833	17,544(23.96)	55,402(76.03)	2.11

**Table 3:** Distribution of atypical lactation curves according to age at first calving

Calving month	19	20	21	22	23	24	25	26	27	28	29	30	31
Negative B	25	54	136	431	1481	2865	2751	1891	685	237	99	51	5
Negative B and C	21	39	90	293	983	1814	1737	1153	432	155	74	39	3
Total	46 (16 %)	93 (14 %)	226 (30 %)	724 (28 %)	2464 (25 %)	4679 (22 %)	4488 (22 %)	3044 (23 %)	1117 (24 %)	392 (28 %)	173 (34 %)	90 (40 %)	8 (19 %)

Based on age at first calving, the percentage of atypical lactation curve is varied and ranged from 0 to 40 % respectively. In this study, the average atypical lactation curve from 18 to 32 month of calving was around 22 %. The rate of atypical lactation curve in a cow which calved before 24 months and especially after 25 months is high. The occurrence of atypical lactation curve which is due to the absence of lactation peak occurred in about 15–42 % [20, 25]. The other factors leading to atypical lactation curve are few numbers of measurements for estimation of model parameters, inadequate distribution of measurements due to time of sampling and missing measurements for lactation curve phases [26].

**Fig. 1:**
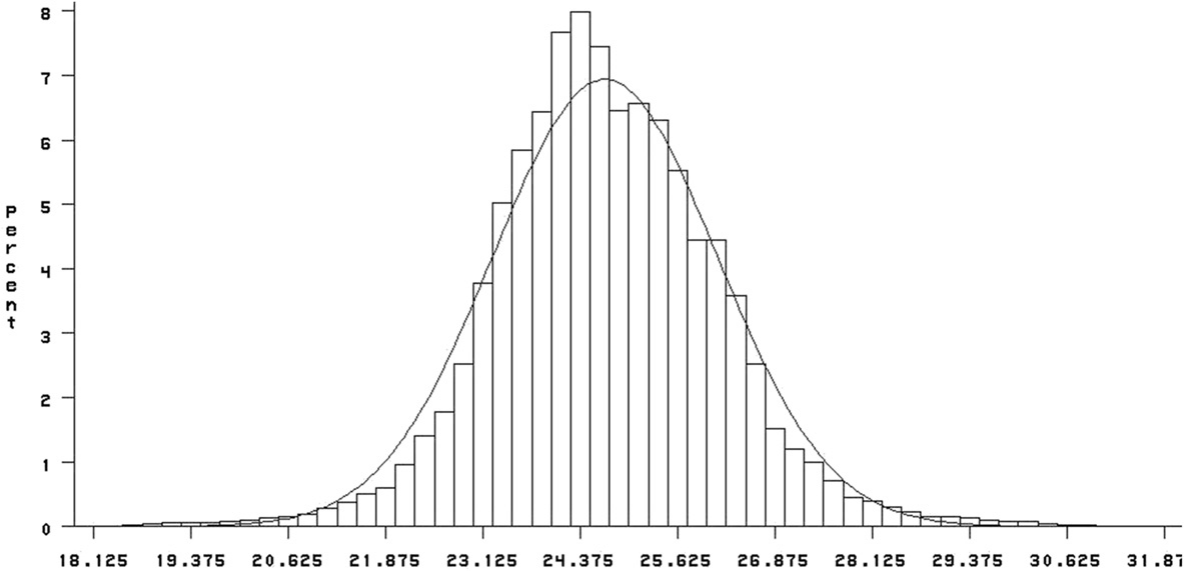
Frequency of age at first calving

Figure 1 illustrates the frequency distribution of age at first calving in our dataset. The proportion of cows which calved before 20 and after 30 months are low. The average age at first calving is 24.65 ± 1.38 (standard deviation) months. This estimation of mean is lower than the estimation of 28.6 months in Spain [27] and 25.9 months in the United States [8], but similar to 24.3 in Irish cattle [28]. The parameters of lactation curve estimated by the Wood model (a, b, c, peak time, peak yield, total milk yields in part1, 2, 3, 305-d, and tow persistency measures) are shown in Table 4. Milk yield increased after calving with mean of 16.62 kg to the peak yield of 33.10 kg that was reached at day 76 and then decreased gradually till the end of 305-d lactation. The average values for the parameters of The Wood lactation curve obtained in present study are almost similar to those reported by Jeretina et al. [26]. They reported that the estimation of the Wood function parameters ± SD (a, b, c, peak time and peak yield) in the first parity of Holstein dairy cows were 15.7 ± 6.7, 0.184 ± 0.149, 0.00290 ± 0.00144, 64.7 ± 25.4 and 26.1 ± 5.1 respectively. The total milk production in part 1, 2 and 3 are 2985.09, 3051.69 and 2681.95 kg respectively. In this study 34.23 % and 35 % of 305-d milk yield were produced in part 1 and part2 respectively. The lowest cumulative milk yield was observed during part3 of lactation (30.7 %).

**Table 4:** Descriptive statistics of lactation curve parameters

Parameters	Mean	SD
a	16.62	6.60
b	0.27	0.03
c	0.003	0.001
Peak time (day)	76.60	24.86
Peak yield (kg)	33.10	5.19
Part1	2985.09	496.87
Part2	3051.69	579.44
Part3	2681.95	647.14
Milk305	8718.73	1623.05
Persis1	102.27	9.68
Persis2	89.82	15.89

The mean of persis1 was higher than persis2 (102.27 vs 89.82). This indicates that persistency of lactation between 101 and 200 days is higher than that of 201 – 305 days. Cakilli and Gunes [29] reported that the ratio of persis 1 and 2 for Brown Swiss cows were 91.93 and 59.91 respectively.

Phenotypic correlations among the lactation curve parameters, production characteristics and 305-d milk yield in different season of calving are presented in Table 5 and 6. From the results, the phenotypic correlation between parameters in spring, summer, autumn and winter ranged from −0.87 to 0.98, −0.86 to 0.97, −0.86 to 0.93 and −0.87 to 0.93 respectively. In all seasons of calving phenotypic correlation between persis1and a and c is negative, but it is positive with the other parameters (b, peak time and peak yield). This means that if cows started lactation with high milk yield, parts1, 2 and 3 and total 305-d milk yield will be high. The maximum phenotypic relationship between initial milk yield and total 305-d milk yield was observed in cows calved in spring (0.40). Although we obtained a moderate phenotypic correlation between initial milk production and 305-d milk yield in this study, Bouallegue et al. [18] reported 0.74 for this relationship in Holstein Friesian of North Africa.

**Table 5:** Phenotypic correlations among parameters of lactation curve in different season of calving (spring = upper diagonal, summer = lower diagonal)

	a	b	c	Peak time	Peak yield	Part1	Part2	Part3	Milk 305	Persis1	Persis2
a		−0.87	−0.60	−0.44	0.27	0.46	0.40	0.34	0.40	−0.23	0.12
b	−0.86		0.78	0.34	0.13	−0.06	0.009 ns	−0.25	−0.11	0.12	−0.28
c	−0.59	0.76		−0.21	0.05	−0.05	−0.30	−0.62	−0.37	−0.51	−0.79
Peak time	−0.41	0.34	−0.22		0.10	−0.03	0.39	0.50	0.33	0.85	0.71
Peak yield	0.23	0.19	0.06	0.18		0.97	0.89	0.66	0.88	0.09	0.03
Part1	0.42	−0.01 ns	−0.05	0.04	0.97		0.86	0.66	0.98	−0.00 ns	0.01 ns
Part2	0.37	0.06	−0.27	0.44	0.90	0.87		0.91	0.94	0.49	0.46
Part3	0.24	−0.18	−0.58	0.54	0.70	0.70	0.92		0.93	0.65	0.74
Milk305	0.36	−0.05	−0.34	0.38	0.89	0.99	0.94	0.89		0.43	0.46
Persis1	−0.24	0.16	−0.49	0.85	0.16	0.05	0.51	0.66	0.46		0.90
Persis2	0.11	−0.24	−0.77	0.70	0.08	0.05	0.47	0.73	0.48	0.90

**Table 6:** Phenotypic correlations among parameters of lactation curve in different season of calving (autumn = upper diagonal, winter = lower diagonal)

	a	b	c	Peak time	Peak yield	Part1	Part2	Part3	Milk 305	Persis1	Persis2
a		−0.86	−0.60	−0.45	0.19	0.39	0.34	0.19	0.32	−0.31	0.07
b	−0.87		0.77	0.36	0.22	0.01	0.12	−0.14	0.00 ns	0.22	−0.22
c	−0.60	0.77		−0.19	0.10	−0.01	−0.19	−0.53	−0.28	−0.43	−0.75
Peak time	−0.48	0.38	−0.17		0.14	0.00 ns	0.40	0.51	0.34	0.86	0.74
Peak yield	0.24	0.16	0.08	0.09		0.97	0.92	0.71	0.90	0.15	0.04
Part1	0.44	−0.04	−0.03	−0.05	0.97		0.88	0.70	0.98	0.02	0.00 ns
Part2	0.37	0.06	−0.23	0.37	0.90	0.87		0.91	0.93	0.48	0.41
Part3	0.25	−0.20	−0.57	0.48	0.68	0.67	0.91		0.90	0.62	0.69
Milk305	0.37	−0.07	−0.32	0.30	0.89	0.98	0.93	0.91		0.42	0.42
Persis1	−0.30	0.21	−0.43	0.86	0.09	−0.02	0.45	0.62	0.40		0.88
Persis2	0.07	−0.22	−0.75	0.72	0.02	−0.01 ns	0.42	0.71	0.42	0.89	

Moreover, cows with higher initial milk yield show higher peak yield and this peak would be early during lactation. Negative phenotypic correlation between a&b indicated that cows with high milk production have long ascending slope and lower rate of descending during lactation. The negative correlation between a and b&c indicate that cows with higher initial milk, reach their peak yield faster, then show a quicker decline after peak of production. The positive correlation between b and c in all seasons of calving implies that cows that peak more rapidly also show more rapid decline after peak. These results are similar to the finding of Gradiz et al. [21]. In this study the phenotypic correlation between peak time and peak yield in different season of calving were positive (between 0.09 and 0.18). Cilek et al. [23] reported that the phenotypic correlation between peak time and peak yield is negative (−0.117). Also Bouallegue et al. [18] reported −0.25 for this correlation in Holstein-Friesian cows in North Africa. In contrary, Farhangfar and Rowlinson [30] found it zero but Boujenane and Hilal [31] and Yamazaki et al. [32] obtained it 0.13 and 0.02 respectively.

The phenotypic correlation between patrt1 and 305-d milk yield is lower that of part 2 and 3 with total milk yield. This means that milk yield is lower in late lactation compared to the first and the mid lactation. The important of part 1 and 2 in proportion of total milk yield are higher than part3. Correlation between peak yield and milk 305-d is higher than peak time and milk 305-d in all seasons of calving. These results suggested that cows with higher peak yield produce higher total milk yield in 305-d lactation and the role of peak yield is completely higher than peak time in prediction of 305-d milk yield. High correlation between peak yield and 305-d milk yield suggesting selection of animals for 305-d milk yield is based on peak yield [33]. Correlation between peak time and persistency measures (persis1 and persis2) is high (0.85 and 0.71; 0.85 and 0.70; 0.86 and 0.74 and 0.86 and 0.72) in spring, summer, autumn and winter respectively. This indicates that as the interval between initial milk yields and peak yield increased, the persistency improved. So it seems that persistency, peak time and peak milk yield are three factors determining the shape of lactation curve. Correlation between different persistency measures and milk 305-d in all seasons of calving which were moderate and positive ranged from 0.40 to 0.48 (Tables 6 and 7). Some persistency measures are negatively correlated with 305-d milk yield [34]. It indicates that selection for higher persistency increased total milk yield. But some researchers ([17, 23, 30, 35] reported positive phenotypic relationship between these traits. A persistency measure that is independent of milk yield will allow more efficient for total yield and persistency simultaneously. Gengler [36] recommended that a good persistency measures should be independent from yield. Solkner and Fuchs [37] mentioned that the relationship between persistency and total milk yield depends on persistency measured used. Persistency measures based on ratios are positively correlated, but measurements based on variation, negatively. Cobuci et al. [38] reported that measuring method of persistency based on sum of milk yield in different lactation periods sometimes present positive correlation regarding total production. Our results showed that correlation between persistency and total milk yield is lower than the correlation between persistency and peak milk yield (Tables 5 and 6) suggesting that peak milk yield is a more important trait in determining the total 305-d milk yield than persistency. This founding is in agreement with the result of Bouallegue et al. [18].

**Table 7:** Least square means (±SE) of parameters of lactation curve in different season of calving

Season of calving	a	b	c	Peak time	Peak yield	Part1	Part2	Part3	Milk 305	Persis 1 %	Persis 2 %
Spring	15.47 (0.33)	0.22 (0.00)	0.003 (0.00)	67.38 (1.25)	29.31 (0.23)	2645.67 (21.31)	2585.91 (24.54)	2190.51 (28.84)	7422.09 (68.75)	98.02 (0.48)	83.15 (0.79)
Summer	15.30 (0.32)	0.22 (0.00)	0.003 (0.00)	69.00 (1.18)	29.30 (0.22)	2644.91 (20.19)	2594.95 (23.25)	2219.49 (27.32)	7459.36 (65.13)	98.21 (0.45)	84.00 (0.75)
Autumn	15.55 (0.32)	0.22 (0.00)	0.003 (0.00)	69.14 (1.20)	29.97 (0.22)	2706.0 (20.41)	2648.56 (23.51)	2251.13 (27.62)	7605.77 (65.85)	97.94 (0.46)	83.35 (0.76)
Winter	15.68 (0.34)	0.21 (0.00)	0.003 (0.00)	67.48 (1.28)	29.88 (0.24)	2683.00 (21.82)	2594.29 (25.13)	2196.36 (29.53)	7473.66 (70.39)	96.90 (0.494)	82.18 (0.81)

**Fig. 2:**
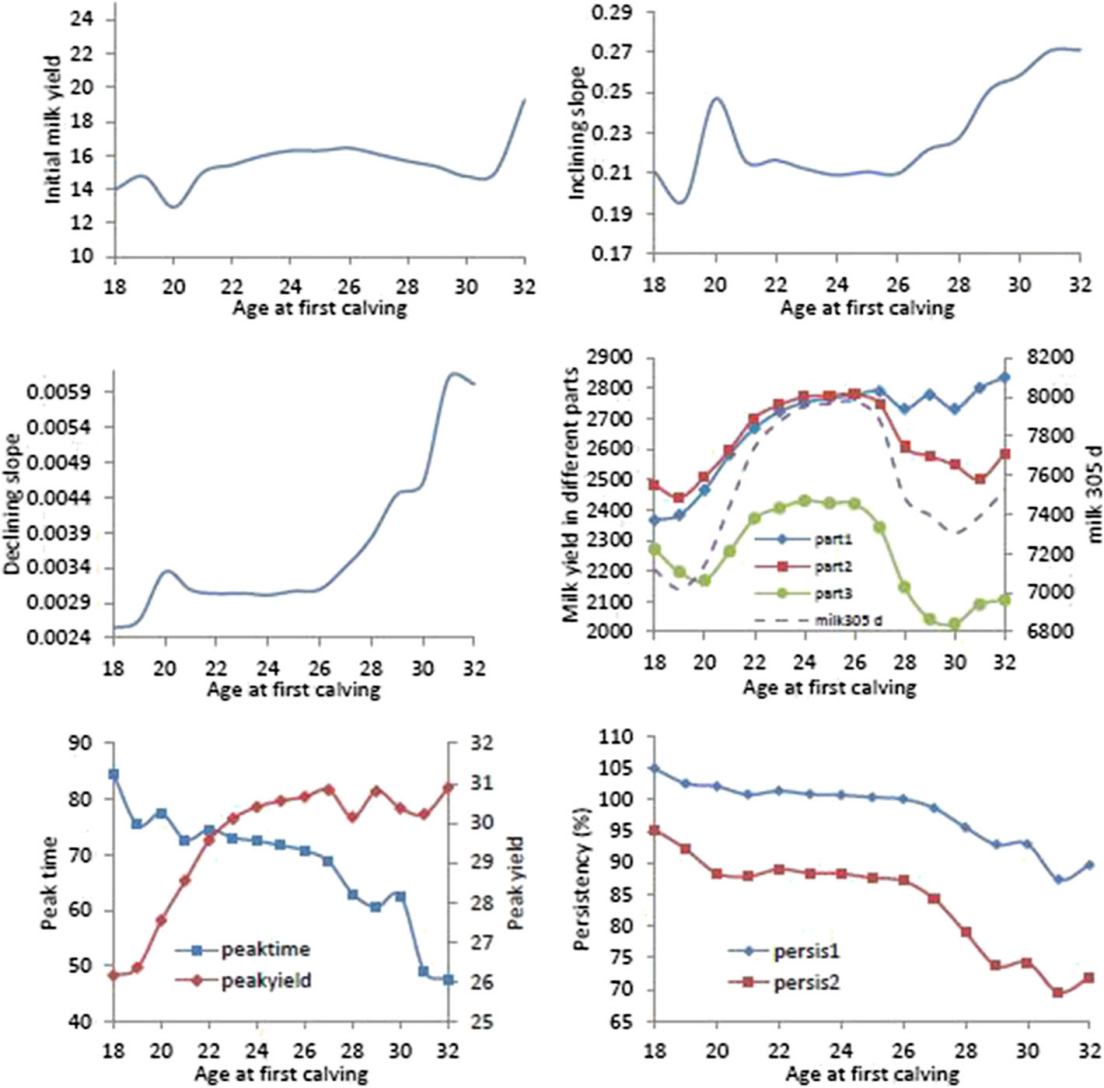
Phenotypic trend of parameters of lactation curve based on AFC in Holstein dairy cows

Least square means of parameters of lactation curve in different age at calving was shown in Fig. 2.

AFC is an important non genetic factor that affects total phenotypic variance of milk production. According to Kume and Tahiri [39] this factor accounts for 30–40 % and 2–5 % of the total variance of milk production at the beginning and end of lactation, respectively. The associations among age at first calving and parameters of lactation curve were significant (*p* < 0.001). The fluctuation of parameters of a, b and c are more at two end of age at first calving. The maximum parameters were obtained at the end of AFC. Based on the results, with increasing age at calving from 18 to 26.2 months, milk yield in part 1, 2, 3 and 305-d increased and then gradually decreased. This means that there is a moderate negative correlation among AFC and part1, 2, 3 and total 305-d milk in primiparous Holstein cattle. This finding is in agreement with the finding of Niloforooshan and Edris [7]. The results from regression analysis on our data showed that 1 month increase in AFC from 18 to 26 months raised 305-d milk yield by around 138 kg and from 27 to 32 months decreased by 61 kg. It seems that a moderate negative effect of increasing AFC on 305-d total milk yield in Iranian primiparous cows was observed after 26 months. This is in contrast with the result of Bewley et al. [40] who reported just a negative effect of increasing AFC on milk yield. They also reported 1 month increasing of AFC was associated with 102.5 kg reduction in total milk yield. But our results are in agreement with finding of Niloforooshan and Edris [7]. Also monthly change of peak time and yield from 18 to 32 months were about −2.09 day and 0.296 kg respectively. The phenotypic trend of other lactation curve parameters from 18 to 32 months of calving (a, b, c, persis1 and persis2) were 0.178, 0.0039, 0.00021, −1.063 and −1.663 respectively. Berry and Cromie [28] reported an increase of 0.2 kg and 55.5 kg/month of peak milk and 305-d milk yield by increasing 1 month of AFC. Ben Gara et al. [41] also proposed that less than 24 month and greater than 26 months of AFC were associated with low 305-d milk yield. Teke and Murat [42] reported that increasing of 305-d milk yield related to increasing AFC is due to development of mammary glands. So the interval between 24 and 26.5 months of calving is the best time of calving in primiparous Holstein for maximizing 305-d milk yield. Ettema and Santos [43] also reported that the optimum AFC is between 23 and 24.5 months of age. Our finding is not in agreement with the results of Heinrichs and Vazquez-Anon [44] who mentioned that 305-d milk yield of heifers which calved at ≥26 months is similar to those calved at 24 months. The increment of AFC from 20 to 40 months is associated with linear increments in 305-d milk yield [17] but our result show that this relationship is quadratic not linear. The phenotypic trend of peak time and peak yield are shown in Fig. 2 too. The Least square mean of peak yield increased slightly from 26 kg in 18 months of calving and increased steadily to 30 kg in 28 months then the reach plateau. The increase in peak yield with the increasing age at calving in dairy cows was consistent with the results reported by Dedkova and Nemcova, [45] and Berry and Cromie [28]. The least square of peak time decreased with increasing of AFC. For example, cows calved in 20 months had peak time around 75 days; whereas, cows calved at 31 months had peak production around 50 days of lactation (Fig. 2). The similar result was reported by Leclerc et al. [46]. Both persistency measures decreased with increasing age at first calving. As shown in Fig. 2, with increasing of AFC, The least square means of persistency decreased. The Least square means of persis1 is higher than persis2 during different AFC. In other words, this means that persistency of lactation between 101– 200 days is higher than that of 201–305 days. Kaya and Kaya [47] reported that the average amount of persis1 and 2 are 84.6 % and 61.5 % in Holstein cattle, respectively. Muir et al. [34] also reported that heifers first inseminated at younger age than average (early AFC) had better persistency than the other heifers.

**Fig. 3:**
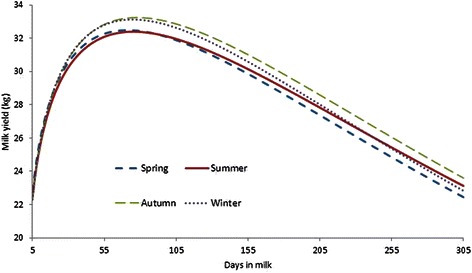
Lactation curves according to different season of calving in Holstein dairy cows

**Fig. 4:**
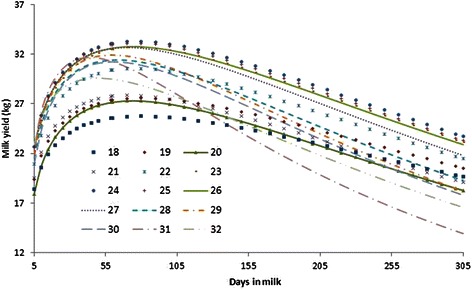
Lactation curves according to different age at calving in Holstein dairy cows

Least square means of lactation curve parameters in different season of calving. For season of calving the lowest a (initial milk yield) occurred for cows calved in summer and it was significant different among least square means of initial milk yield in spring, summer, autumn and winter. The highest parameters are attributed to winter and autumn, respectively (15.68 and 15.55). Least square means of inclining and declining slope of lactation curve were almost similar and non-significant in all season of calving. Similar results were reported by Tekerli et al. [25]. Considering the season of calving, peak time happened earlier in those cows calving in spring and winter, compared with those calving in summer and autumn (Table 7). The highest peak yield was associated with cows calved during autumn (29.97 kg) and winter (29.88 kg) but the lowest peak milk yield was related to the cows calved in spring (29.31 kg) and summer (29.30 kg) respectively. Probably the reason of this difference is mainly related to high environmental temperature in Iran. In an opposite report, higher peak yield was observed by Schei et al. [48] for spring calving Red Cattle compared to autumn calving cows. Many studies [13, 25, 49] reported the significant effect of the season of calving on total 305-d milk yield. The results indicate that the cows calved in autumn and winter produced more 305-d milk yield than that of cows calved in spring and summer (Table 7). These findings agree with results reported by Madani et al. [50] and Kaya et al. [11]. With respect to positive phenotypic correlation between initial milk yield and 305-d milk production in different calving seasons, it is concluded that initial milk production and peak yield are the most important parameters of lactation curve in determining the total lactation yield (Tables 5, 6 and 7). Moreover, the maximum proportion of 305-d milk yield is attributed to part 1 followed by part 2 and part3. Autumn and winter calving cows had higher yield at first and mid lactation compared to cows calved in other seasons. The most important environmental or non-genetic effects are the stage of lactation [1], age at calving, parity, calving season [2, 3] pregnancy stage [4] and the common herd effect. The basic criteria for defining calving season are the feeding as well as temperature and humidity of local area. Seasons with high temperatures have negative influence on milk traits, mainly fat content. Calving season causes differences in milk production and in the shape of lactation curve. Figure 3 shows the lactation curve with respect to calving season. This figure also indicated that the shape of lactation curve is largely dependent on the season of calving. Also mean peak yield of cows that calved in winter and autumn are higher than that cows which calved in summer and spring. Moreover, based on Fig. 3, the level of milk production of cows which calved in autumn and winter is higher than the other two seasons. Winter and autumn calving cows produce slightly higher milk yield during lactation. Olori and Galesloot [51] reported the calving season affected on the shape of lactation curves of dairy cattle in Ireland. in the first lactation Czech Holstein cows, Dedkova and Nemcova [45] observed that winter calving cows had higher milk production. Lactation curve of the primiparous cows, based on age at calving, are presented in Fig. 4. The best performance of lactation yield and persistency is related to the cows which calved in 24, 25 and 26 months. The level of milk production of cows which calved around > 26 and < 23 months is not optimal. In other words, the highest daily milk yield was reached by cows around 24 and 26 months of age. This is in agreement with the finding of Niloforooshan and Edriss [7] who suggested that cows which calved older than 24 months showed lower milk yield and delaying of age at first calving can increase rearing costs in these cattle. Cows calving younger have lower production, especially in the first lactation [13]. The worst performance and persistency is related to cows that calved in 31 and 32 months.

Ettema and Santos [43] reported the least square means of milk yield of primiparous Holstein cows that calved in three different age groups (low,≤ 23 month; medium = 23–24.6 month and high,≥ 24.6 month). They reported that milk production for all three group were similar but cows in low group, produce less milk than those in the other groups especially after 50 DIM. This result is completely different with our finding maybe due to more different age at calving classes. Moreover, milk production in cows calved in 18–21 month was completely different with those which calved in 24–26 month from the onset of lactation.

Heritabilities for lactation curve traits and at first calving are presented in Table 8. The point heritability estimates ranged from 0.021 to 0.011. Also, peak yield, part1, 2, and milk 305-d showed the highest heritability. These results are in agreement with those reported by Boujenane and Hilal [31]. They found that the heritability of a, b, c, peak time, peak yield, persistency and milk 305-d are 0.02, 0.05, 0.02, 0.06, 0.10, 0.05 and 0.08 respectively. But Yilmaz et al. [52] reported higher heritability for these traits in Brown Swiss cattle (0.14, 018, 0.15, 0.42, 0.23, 0.29 and 0.25 respectively).

**Table 8:** Estimates of heritabilities ± SE of lactation curve parameters and AFC

Parameters	a	b	c	Peak time	Peak yield	Part1	Part2	Part3	Milk 305-d	Persis1	Persis2	AFC
Heritability	0.021 (0.005)	0.015 (0.004)	0.024 (0.011)	0.022 (0.005)	0.115 (0.011)	0.105 (0.010)	0.108 (0.010)	0.072 (0.008)	0. 109 (0.012)	0.026 (0.005)	0.022 (0.005)	0.075 (0.002)

**Fig. 5:**
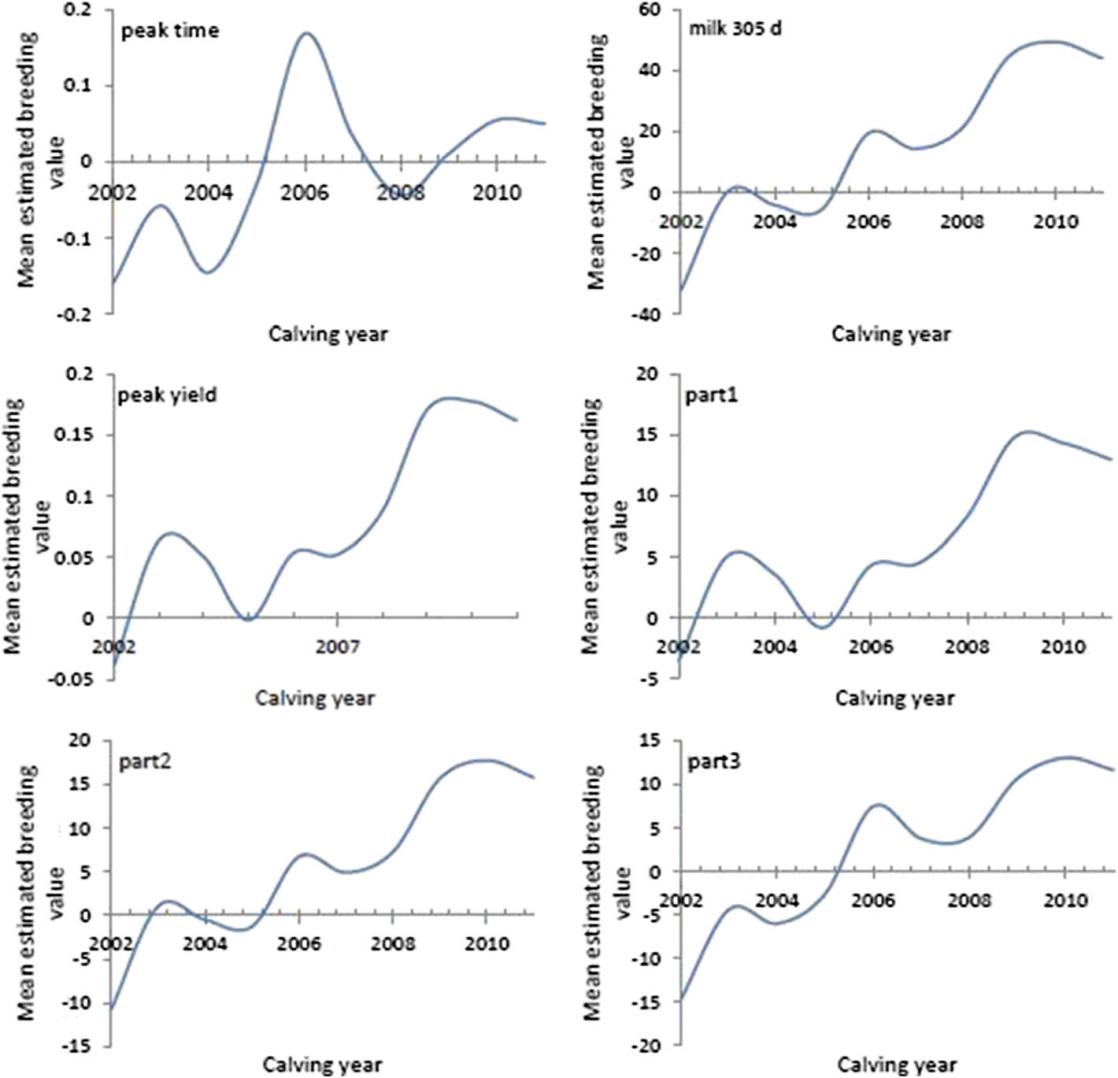
Mean estimated breeding values for some parameters of lactation curve in Holstein dairy cows

Difference between heritabilities, reported by researchers, could due to differences in statistical models and genetic variation between populations plus variation of the breeds in different environmental conditions [53]. The estimation of heritability for persistency measures and age at first calving are low (Table 8). Low to medium heritability amount of different persistency measures were reported by Atashi et al. [35] and Boujenane and Hilal [31]. Different estimation of heritability reported in AFC ranged from less than 10 % to more than 30 % [41]. Our results are in agreement with finding of Muir et al. [34]. Heritabilities of persistency, peak time, a, b, and c are low so these traits are not good indicators for genetically improvement of the lactation curve shape. The low heritabilities for lactation curve traits and AFC indicate that such traits were mainly affected by environmental variation, these traits have no considerable genetic variation. Faraji-Arough et al. [54] and Ben Gara et al. [41] reported low heritability for AFC (no benefit selection for improvement of this trait in dairy cows) but higher estimation of heritability for AFC in African Holstein cattle (0.24) indicate that this trait can be improved genetically through selection [53]. These discrepancies may reflect variations in the models, breeds, populations, lactation curve, data editing and sampling errors. The study of genetic trend in populations is a useful tool for monitoring of selection strategies and it permits visualization of the efficiency of selection procedures [55]. The precision of genetic trend estimation is enhanced greatly as the number of increases [56]. The trend of estimation breeding values (EBV_S_) for peak time and yield, part1, 2, 3 and 305-d milk yield calculated according to calving year by animal model method, according to calving year, are presented in Fig. 5. There was an increasing trend for peak yield from 2002 to 2011 but for peak time the maximum EBVs was obtained in 2006 and the fluctuation of EBVs in this trait is higher than that of peak yield. Estimated breeding values for peak time and yield ranged from −0.016 to 0.05 and −0.03 to 0.17 respectively.

The fluctuations in these parameters were sharper than EBVs of part1, 2, 3 and 305-d because environmental effects can easily influence on lactation curve milk yield. Genetic trends of part1, 2, 3 and 305-d milk yield showed a substantial tendency among different calving years and almost the positive trend was detected after 2005 and the highest mean EBVs was found in the last year. Generally, genetic trends for part1, 2, 3 and 305-d milk yields were positive and estimated to be 1.80 ± 0.35, 2.79 ± 0.36, 2.77 ± 0.37 and 8.13 ± 1.01 kg/year respectively. A genetic improvement in these traits was found between 2002 and 2011 in primiparous cows; it indicates an increasing of breeding values and selection up on better sires and dams in this period and finally the positive values for regression coefficients suggest genetic improvement in the farms for 305-d total milk yield. The genetic trend for 305-d milk yield in our study was similar to the 7.0 kg/year reported by Lôbo et al. [57] but lower than the 13.88 kg/year obtained by Verneque et al. [58]. Katok and Yanar [55] reported that estimated annual genetic and phenotypic trends were 3.73 ± 4.07 and −17.73 ± 9.64 kg for 305-d milk yield of Holstein Frisian cows in Turkey but Hallowell et al. [59] and Osman et al. [60] found a positive trend in EBV 19 and 18.97 kg/year for the first lactation milk yield in South African Ayrshire and Holstein cattle, respectively. In another study Ramatsoma et al. [61] reported a quadratic regression line for describing the genetic trend in milk yield over a specific period. The lower genetic trend for 305-d milk yield in our study may be due to smaller estimation of 305-d milk yield heritability.

## Conclusion

Production performance of dairy cows affected by some factors including age at first calving, parity, calving and production season, stage of lactation and common herd effect. The frequency of atypical lactation curve were affected by seasons of calving and age at calving as the probability of occurrence of atypical lactation curve were the highest in spring & summer and the lowest in autumn & winter respectively. The rate of atypical lactation curve is in consistent when AFC is during 24 to 26 months. Cows with higher peak yield produce higher total milk yield in 305-d lactation and the role of peak yield is completely higher than peak time and persistency in prediction of 305-d milk yield. The best performance of lactation yield and persistency is related to the cows which calved in 24, 25 and 26 months. The low heritabilities for lactation curve parameters and AFC indicate that such traits are mainly affected by environmental variation. Moreover, these traits have no considerable genetic variation. Genetic trends for 305-d milk yields were positive and estimated to be 8.13 ± 1.01 kg/year.
